# Inclusion of polysaccharides in perovskite thin films: from in-solution interaction to film formation and stability†

**DOI:** 10.1039/d4na01036a

**Published:** 2025-03-11

**Authors:** Francesco Bisconti, Antonella Giuri, Nadir Vanni, Sonia Carallo, Silvia Spera, Rosamaria Marrazzo, Riccardo Po', Paolo Biagini, Barbara Paci, Amanda Generosi, Marco Guaragno, Carola Esposito Corcione, Andrea Listorti, Silvia Colella, Aurora Rizzo

**Affiliations:** a CNR NANOTEC – Istituto di Nanotecnologia c/o Campus Ecotekne, Via Monteroni 73100 Lecce Italy antonella.giuri@nanotec.cnr.it aurora.rizzo@nanotec.cnr.it; b Dipartimento di Matematica e Fisica “E. De Giorgi”, Università del Salento Campus Ecotekne, Via Arnesano 73100 Lecce Italy; c Research Center for Renewable Energy & Environmental, Istituto Donegani, Eni S.p.A. Via Fauser 4 I-28100 Novara Italy; d CNR-ISM-SpecX Lab Via del Fosso del Cavaliere 100 Roma Italy; e Dipartimento di Ingegneria dell'Innovazione, Università del Salento Via per Monteroni, km 1 73100 Lecce Italy; f Dipartimento di Chimica, Università di Bari Via Orabona 4 70126 Bari Italy; g CNR NANOTEC – c/o Dipartimento di Chimica, Università di Bari Via Orabona 4 70126 Bari Italy

## Abstract

Despite perovskite solar cells (PSCs) being among the most promising photovoltaic technologies, their widespread adoption requires further advancements in material processability and long-term stability. Polysaccharides have emerged as effective additives for assisted perovskite thin film crystallization in one step dripping-free deposition. Here, with the aim of rationalising their effect, the role of the hydroxyl groups (–OH) in the polymer structure, affecting the formation of perovskite–polymer nanocomposites, has been thoroughly analysed by comparing two celluloses, hydroxyethyl cellulose (HEC) and cellulose acetate (CAT), in which some of the –OH groups are replaced by acetyl groups. The combination of nuclear magnetic resonance (NMR), differential scanning calorimetry (DSC), thermogravimetric analysis (TGA) and rheological analysis showed that HEC strongly interacts with perovskite precursors already in solution, retards DMSO evaporation and then modifies the crystallisation dynamics process, resulting in a film characterised by highly uniform grain structure and improved film stability, with a power conversion efficiency (PCE) of 15.89%. In contrast, CAT with partially substituted –OH groups showed weaker interactions resulting in non-uniform crystal growth and poor film morphology. Furthermore, Energy Dispersive X-ray Reflectivity (EDXR), Atomic Force Microscopy (AFM), and X-ray Diffraction (XRD) confirm that HEC-based films maintain structural stability under light-aging conditions, whereas pristine methylammonium lead iodide (MAPbI_3_) undergoes significant degradation. These findings highlight the potential of HEC as an intrinsic stabilizer for perovskite films, paving the way for more durable and scalable PSC technologies.

## Abstract 2

The –OH groups in cellulose chains allow controlling perovskite crystallization, thus performance, and stability when cellulose is used as an additive to deposit perovskite films in a single-step, as shown by replacing the –OH with acetate groups.

## Graphical abstract

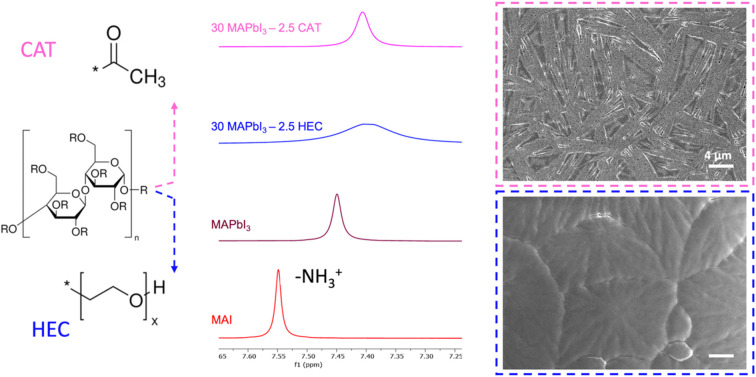

## Introduction

Solar cells are a favorable technology to harvest and convert solar light into energy, representing a valid countermeasure to fossil fuel depletion and energy demand increase.^1,2^ Metal halide perovskites (MHPs) have emerged in recent years as an exciting class of semiconductors due to their interesting optoelectronic properties, such as high carrier mobility, strong light absorption and tunable band gap. MHP-based solar cells have garnered significant scientific interest,^3^ achieving a remarkable increase in power conversion efficiency (PCE) from 3.8% to over 27% as a standalone technology within a decade, making them competitive with silicon-based photovoltaics. Furthermore, in tandem configurations with silicon, they have surpassed 34% efficiency.^4–10^

Despite these fascinating properties and rapid advances, several issues must be overcome before reaching the technological maturation for commercialization, above all, the reproducibility of the material deposition and its operational stability. More in detail, the stability issue of metal halide perovskite-based materials and devices can be associated with intrinsic factors like hygroscopicity, ion migration, thermal instability, and extrinsic (environmental) factors like moisture and oxygen.^11–17^

Among the strategies adopted to solve the stability issue of perovskites there are several “internal” encapsulation methods based on the use of cation mixing^13,18–20^ or small molecules as additives.^21–27^ Natural polymers, extensively studied in various research fields, from biomedical^28–30^ to food packaging technologies,^31–33^ also gave their contributions for perovskite,^34–39^ improving the film quality and reducing the ion migration at the grain boundaries, as well as realizing interface encapsulation which decrease the defect density, or preventing leakage of lead.^40,41^

Then, strategies based on external encapsulation rely on the use of glass or polymeric layers that act as an encapsulant, for the external surface of the entire photovoltaic device, to directly hinder the infiltration of moisture and oxygen, preventing its damage.^42,43^ Obviously, dealing with an intrinsically robust perovskite, obtained through the use of internal encapsulation methods, is a more effective strategy towards long term operational stability. In this context, first control of the perovskite material crystallization *via* wet processing has proved to be a successful approach to control the final film properties. In detail, the interaction between the different components, that already occurs in solution between the additives and the perovskite precursors, allows the formation of a good quality film through a complex self-assembly process driven by weak interactions of ionic species.^44,45^ This mechanism can also be realized without the use of an additional solvent dripping step, simplifying the fabrication process.^46–48^ In addition, the presence of polymeric additives known to localize at the grain boundaries contributes to improving the film's mechanical strength as well as its thermal stability, without interfering with the film's optoelectronic properties. In our previous work we have extensively studied the influence of different additives on the crystallisation of MHPs with the aim of improving perovskite performance for real life application of perovskite solar cells.^35,46,49^

We have found that different functional groups, that characterize specific polymers, interact differently with the precursors, influencing the final properties of the film.

Among the explored polymeric additives, we found that polysaccharides are able to modify the rheology of the perovskite solution when the precursors are dissolved in dimethyl sulfoxide (DMSO), as a function of precursors and polymer concentration. In this way, easy tunability of the perovskite ink viscosity can be obtained, playing a key role in the scaling up process, where different deposition or printing techniques require specific characteristics of the ink.^35,50–54^

However, despite polysaccharides having an apparently similar molecular structure, they behave differently depending on the functional groups on their structure. In this work we deeply analyse the role of HEC in perovskite film crystallization, with the aim of correlating it with the final film properties, through comparison with cellulose acetate (CAT), where some of the –OH groups are substituted by acetyl groups.

Starting by the study carried out on the solution, NMR analysis revealed the interaction between –OH groups and MAI, that in combination with the increased viscosity of the precursor solution, plays a key role in delaying the evaporation of DMSO, then the crystallization process of the perovskite as observed by DSC and TGA analysis. This results in the formation of a film with closely packed grains organized in the Liesegang rings with respect to the poorer film quality of the MAPbI_3_–CAT composite. Additionally, the higher enthalpy of crystallization observed for the composite with HEC indicates greater film stability, as further demonstrated by monitoring bulk and interface modifications through energy dispersive EDXR, AFM, and XRD during aging study under light/dark cycles.

The performance of the obtained films was further evaluated by incorporating them in an optimised p–i–n device architecture consisting of indium tin oxide (ITO)/poly[bis(4-phenyl)(2,4,6-trimethylphenyl)amine] (PTAA)/MAPbI_3_/[6,6]-phenyl-C61-butyric acid methyl ester (PCBM)/bathocuproine (BCP)/silver (Ag), which shows the best combination of polymer/precursor ink wettability (on the organic hole transported layer, PTAA) during single step deposition and energy level matching, as reported in our previous work.^55^ The best PCE of 15.89% was achieved by MAPbI_3_-HEC based solar cells, compared to the 0.36% of MAPbI_3_-CAT.

Thus this work demonstrates the crucial role of the functional groups of polymeric additives in modulating perovskite crystallization, performances and stability.

## Experimental section

### Materials

Lead(ii) iodide (PbI_2_, ultradry 99.999% metals basis) and methylammonium iodide CH_3_NH_3_I (MAI) were purchased from Alfa Aesar and Dyesol, respectively. Dimethyl sulfoxide anhydrous, 99.9% (DMSO), toluene anhydrous, 99.8% (TOL), chlorobenzene anhydrous, 99.8% (CB), 2-propanol (IPA), PTAA [poly(bis{4-phenyl}{2,4,6-trimethylphenyl}amine)], bathocuproine, 96% (BCP), 2-hydroxyethyl cellulose (HEC) and cellulose acetate (CAT) were purchased from Sigma-Aldrich. [6,6]-Phenyl C_61_ butyric acid methyl ester (PCBM) was purchased from Nano-c.

### Thermogravimetric analysis (TGA)

The analysis was conducted using a TGA SDT Q600, by heating about 10 g of each sample from room temperature to 900 °C at a rate of 10 °C min^−1^ in a N_2_ atmosphere. The polymers were tested before and after a drying process in an oven at 60 °C for 5 days.

The solutions of perovskite precursors, with and without polymers, were tested under the same conditions specified above.

Three measurements were performed on each sample. The onset temperature, Tonset, was calculated by extrapolating the intersection point between the tangent lines to the curve at the beginning of the weight loss.

### Perovskite precursors : polymer based solution preparation

All the solutions were prepared in a N_2_ filled glove-box. In detail, the perovskite precursors were solubilized in DMSO with an equimolar stoichiometry (MAI : PbI_2_ = 1 : 1) and a precursor concentration of 30 wt% at 80 °C for 30 min. Subsequently, a certain amount of each polymer was added to perovskite precursors followed by stirring at 80 °C for 5 h. The formulations are labelled as *x*MAPbI_3_-*yZ*, where *x* is the perovskite precursor concentration (wt%) in DMSO, while *y* is the concentration (wt%) of the polymer (*Z*) related to perovskite.

### Differential scanning calorimetry (DSC)

DSC scans were performed on perovskite precursor solutions using a DSC (Mettler Toledo 622). About 3 μl of each solution was dropped into a sealed pan with a perforated lid, in order to allow the evaporation of solvents and by-products. The samples were heated from 25 up to 250 °C at 10 °C min^−1^ scan rate under nitrogen flow (80 ml min^−1^). Three measurements were performed on each sample. The onset temperature, Tonset, was calculated by extrapolating the intersection point between the tangent lines to the curve at the beginning of the reaction. The crystallization enthalpy, *H*_c_, was deduced from the area of the respective exotherms based on an extrapolated horizontal baseline aligned to the asymptotic value of the DSC signal at the end of the reaction.

### Rheological tests

A Malvern Kinexus Pro+, using parallel plate geometry (25 mm radius), was employed to investigate the rheological behaviour of the inks. The measurements were performed at 23 °C analyzing the solution viscosity in a shear rate range of 10–1000 s^−1^.

### NMR analysis

^1^H-NMR spectra have been recorded with a Bruker Avance 400 NMR instrument, operating at 400 MHz.

The chemical shift reference is the DMSO signal, at 2.56 ppm. Spectral parameters are: 90° pulse, 10 ppm of spectral width, 128 scans and 1 s of delay between scans.

T_2_ measurements have been performed with the sequence “T_2_CPMG” from the Bruker library.

### Device fabrication

MAPbI_3_-polysaccharide composite films were integrated in p–i–n geometry planar solar cells fabricated by spin coating, using glass substrates with ITO as the anode. The device architecture was selected based on the optimised device layout from our previous work.^55^ PTAA was used as the hole transporter layer, and PCBM and BCP were selected as the electron transport layer and hole blocking layer, respectively. The optimised BCP-PCBM coupling has several key functions, including reducing the energy barrier between the PCBM and the Ag electrode, allowing for more efficient electron transport; minimising charge recombination; improving stability as BCP prevents Ag diffusion into the perovskite layer, a known degradation pathway, thus increasing device lifetime.^56,57^

The deposition parameters were: 6000 rpm for 30 s followed by 100 °C × 10 min annealing for 1.5 mg ml^−1^ toluene PTAA solution, 1000 rpm for 60 s for 25 mg ml^−1^ chlorobenzene PCBM solution and 6000 rpm for 20 s for 0.5 mg ml^−1^ 2-propanol BCP solution. All materials were deposited in a N_2_ filled glove box (<0.1 ppm [O.2], < 0.1 ppm [H_2_O]) except for PTAA. 80 nm Ag was thermally evaporated (Lesker Co. instrument) in high vacuum (5 × 10^−6^ mBar) as the cathode top electrode, with a deposition rate of 0.6 A s^−1^ and employing a mask that defines a 0.04 cm^2^ active area. The photovoltaic device *J*–*V* characteristics were acquired in a N_2_ atmosphere at 25 °C using a Keithley 2400 Source Measure Unit under an irradiation intensity of 100 mW cm^−2^, employing an Air Mass 1.5 Global (AM 1.5 G) Solar simulator (Newport 91160A, periodically calibrated lamp).

### Perovskite film deposition

Perovskite thin film deposition was realised by spin coating on glass substrates (25 × 25 mm^2^). First, a layer of poly[bis(4-phenyl)(2,4,6-trimethylphenyl)amine] (PTAA) was deposited under ambient air conditions by spin coating (6000 rpm for 20 s followed by 100 °C for 10 min of annealing) on the glass substrate. Then, reference MAPbI_3_, MAPbI_3_-HEC and MAPbI_3_-CAT samples were deposited in a nitrogen filled glove box with the following parameters: 4000 rpm for 25 s (toluene antisolvent step at 15th sec.) followed by 100 °C for 15 min of annealing for reference perovskite, 12 000 rpm for 60 s followed by 100 °C for 45 min of annealing for both HEC and CAT based perovskite. The as prepared samples were then characterized as described below.

### XRD measurements

X-ray diffraction measurements (XRD) were performed in reflection mode on a panalytical empyrean diffractometer (Cu-anode: K-Alpha1 [Å] = 1.54060; K-Alpha2 [Å] = 1.54443). The Bragg Brentano configuration was used as an incident optical pathway focusing the impinging beam with fixed divergent slits (1/32°–1/16°). Detection was accomplished by means of a solid-state hybrid Pix'cel 3D detector, working in 1D linear mode. The sample structure was explored in the range 3° < 2*θ* < 70° with a step size.

### Scanning electron microscopy (SEM)

The SEM analysis was conducted using a Carl Zeiss Auriga40 Crossbeam (Zeiss) instrument in high vacuum and high-resolution mode, equipped with a Gemini column and an integrated high efficiency in-lens detector. A 5 kV voltage acceleration was applied.

### AFM measurements

Images on different portions of the samples were collected by means of an in house developed Atomic Force Microscope (AFM) mounting a 30 μm × 30 μm scanner. Measurements were performed in non-contact mode by means of TAP300Al-G tips, Si probes with an aluminum reflex coating, resonant freq. 300 kHz and force constant 400 N m^−1^.

### Energy dispersive X-ray reflectivity (EDXR)

EDXR measurements were performed by means of a non-commercial in house patented energy dispersive reflectometer in order to study the morphological characteristics of the layered samples, *i.e.* thickness and surface/interface roughness. A W-anode X-ray lamp (10–50 KeV) was used to produce the Bremsstrahlung radiation focused onto the sample by means of micrometric W-slits (20 × 20 μm aperture). A Ge-single crystal solid state detector accomplished detection counting the incoming photons and discriminating their energy. The geometry of the experimental apparatus was kept fixed during measurements, with no angular movement being required and pattern acquisition occurring simultaneously in the whole explored energy-range.

### FTIR-ATR analysis

Fourier Transform Infrared (FTIR) spectroscopy analysis in Attenuated Total Reflectance (ATR) mode was carried out using an IR Nicolet N10 microscope, equipped with a Ge tip and MCT refrigerated detector, based on 256 scans and a 2 cm^−1^ resolution.

## Results

Methylammonium lead iodide perovskite, characterized by a single and stable tetragonal phase at room temperature, represents one of the target materials for solar cell applications^58,59^ and it was used here to explore the role of the polymer inclusion in the final thin film. The developed inks consist of DMSO as the solvent because it is among the safer solvents explored for the perovskite precursors^60,61^ and it is able to strongly interact with perovskite precursors by forming a stable adduct intermediate which contributes to controlling the crystallization process, if properly assisted by a specific method (*e.g.* an anti-solvent, an additive, *etc.*).^62–64^

We then selected a perovskite film without polymeric additives, processed using antisolvent engineering, as the reference. This choice was made based on the well-known challenges^65–67^ in depositing perovskite films without both polymer additives and the antisolvent dripping step, which typically result in significant voids.

Thus, before the inclusion into the perovskite precursor solution, both polymers, HEC and CAT, were dried for 5 days in order to lower the water concentration that could interfere with perovskite crystallization,^68^ reaching values around 1% for HEC and 2% for CAT. This was revealed by the first step of weight loss observed with thermogravimetric analysis (TGA), which is related to the water evaporation process (Fig. S1 and Table S1†). It is also evident that the Tonset of the second weight loss step, ascribed to the thermal degradation of the polymers, is higher than the temperature used for the preparation of the solutions and for the annealing of the perovskite films. This is a first indication that the investigated polymers are compatible with the processing conditions of the perovskite–polymer nanocomposite.

**Fig. 1:**
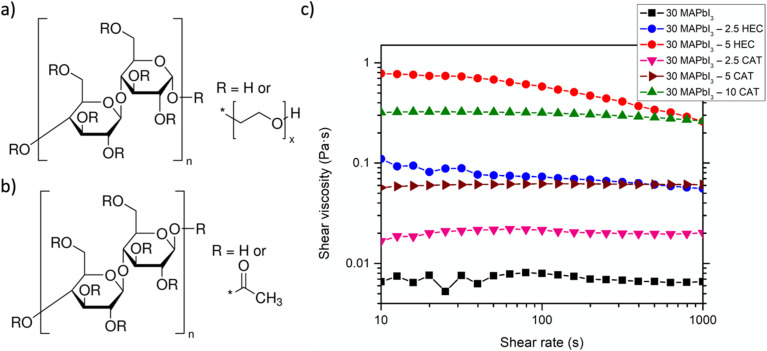
Polymer structure and solution rheology. (a) 2-Hydroxyethyl cellulose and (b) cellulose acetate molecular structure, besides the influence (c) on solution viscosity as a function of shear rate.

Then, the influence of the polymer on the solution rheology was evaluated by measuring the viscosity as a function of shear rate and polymer concentration. As visible from Fig. 1, the 30 MAPbI_3_ solution showed a Newtonian fluid behavior, with a viscosity of 0.006 Pa s that is independent of shear rate. The addition of a small quantity of HEC (2.5 wt%) results in a pseudoplastic-like behavior, with a viscosity of 0.09 Pa s and 0.055 Pa s at low and high values of shear rate. The viscosity increases up to 0.78 Pa s at low values of shear rate and 0.22 Pa s at high values with 5 wt% of HEC. On the other hand, the addition of 2.5 wt% of CAT resulted in a Newtonian behavior, with an increased viscosity of 0.02 Pa s, respect to MAPbI_3_ solution, which increases to 0.06 Pa s with 5 wt% of CAT, still retaining a Newtonian behavior, meanwhile with 10 wt% a pseudoplastic behavior with a viscosity of 0.32 and 0.25 Pa s at low and high values of shear rate, respectively, was observed.

The rheological screening showed a different influence of the two investigated celluloses, which may be the result of both polymer molecular weight and, particularly, polymer functional groups.

To shed light on the mechanism leading to the different behaviours of the two celluloses, we in depth investigated the system, starting with NMR spectroscopy. The evaluation of the T_1_ and T_2_ relaxation times by NMR represents a good approach to assess if an interaction occurs between perovskite precursors and polymers.

**Fig. 2:**
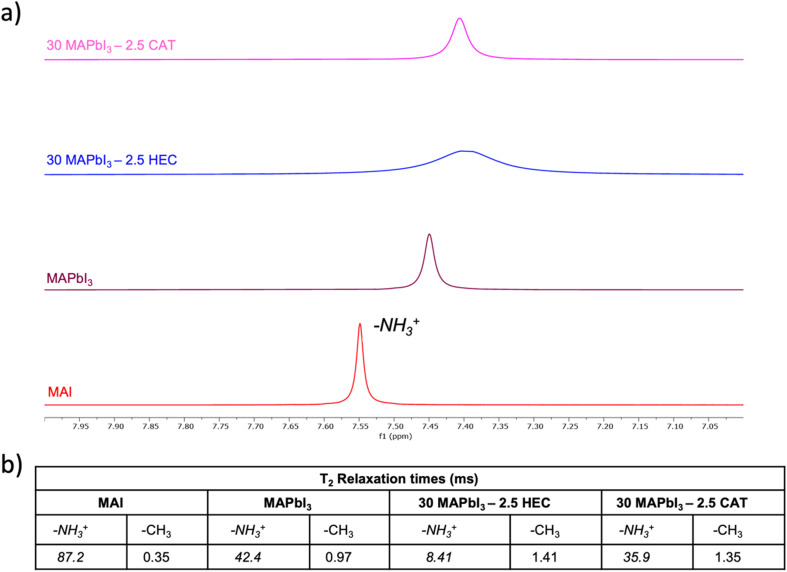
Nuclear magnetic resonance analysis. (a) ^1^H-NMR spectra of 30 MAPbI_3_ – 2.5 CAT, 30 MAPbI_3_ – 2.5 HEC, reference MAPbI_3_ and MAI samples in DMSO-d_6_ focused on –NH_3_^+^ shift. (b) –NH_3_^+^ and –CH_3_ T_2_ relaxation times of 30 MAPbI_3_ – 2.5 CAT, 30 MAPbI_3_ – 2.5 HEC, MAPbI_3_ and MAI samples in DMSO-d_6_ measured by Carr–Purcell–Meiboom–Gill (CPMG) sequence.

As shown in Fig. 2, switching from precursors alone, to precursors in the presence of the polymer, a line broadening is observed in the –NH_3_^+^ signal, which depends on a decrease in T_2_.

A detailed analysis of T_2_ relaxation times reveals distinct interaction mechanisms between the polymers (HEC and CAT) and the perovskite precursors. For MAPbI_3_ doped with HEC, the –NH_3_^+^ group shows a significant reduction in T_2_ (8.41 ms) compared to pristine MAPbI_3_ (42.4 ms) (Fig. 2b). This suggests strong intermolecular interactions, likely hydrogen bonding, between the free hydroxyl groups of HEC and the ammonium group. These interactions enhance spin–spin relaxation by restricting molecular motion, as evidenced by the line broadening observed in the NMR spectra. In contrast, MAPbI_3_ with CAT exhibits a less pronounced reduction in T_2_ (35.9 ms for –NH_3_^+^) (Fig. 2b), indicating weaker interactions. This can be attributed to the partial substitution of hydroxyl groups in cellulose acetate, which reduces the polymer's ability to form hydrogen bonds. Our previous work^69^ has shown that hydrogen-bonding capacity is closely linked to the polymer structure: the abundant –OH groups in HEC enable stronger bonding with –NH_3_^+^, while the acetylated groups in CAT hinder such interactions.

The stronger hydrogen bonding in HEC also affects perovskite crystallization. By interacting with crystal edges through –NH_3_^+^, the polymer imposes spatial constraints during growth, further shortening T_2_. On the other hand, the limited bonding capability allowed by CAT results in less disruption to crystallization dynamics, leading to a smaller reduction in T_2_. These findings highlight how the specific characteristics of the polymers influence their interactions with perovskite precursors and, consequently, the material properties.

**Fig. 3:**
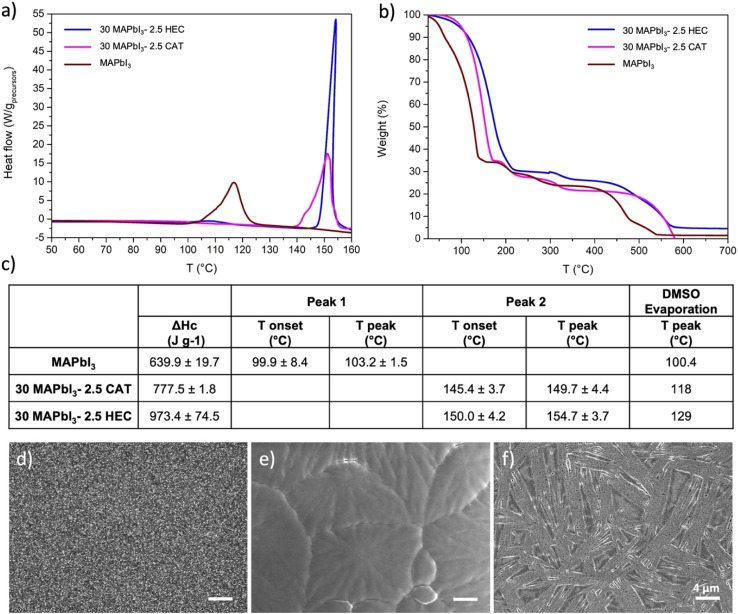
From solution to film morphology. (a) DSC and (b) TGA measurements of reference MAPbI_3_, 30 MAPbI_3_–2.5 HEC and 30 MAPbI_3_–2.5 CAT, along (c) values extracted from TGA; SEM analysis of (d) reference MAPbI_3_, (e) 30 MAPbI_3_–2.5 HEC and (f) 30 MAPbI_3_–2.5 CAT, scale bar: 4 μm.

With the aim of better understanding how the different interactions with the functional group of the polymer influence the perovskite crystallization from solution, dynamic thermal Differential Scanning Calorimetry (DSC) and TGA measurements were performed as a function of the polymer nature as shown in Fig. 3a and b. As already observed in the previous studies,^70^ by comparing the thermograms of the MAPbI_3_ perovskite with that of the DMSO solvent alone, from the two opposite phenomena that occur during the measurement (*i.e.* the endothermic solvent evaporation, and the exothermic perovskite crystallization), an intense exothermic peak at 107.7 ± 7.9 °C, with an onset at 99.9 ± 8.4 °C, associated with the perovskite formation, was predominant in the perovskite solution. The presence of the polymers delays the perovskite crystallization, as already observed in the previous work, showing an averaged value of the peak at 149.7 ± 4.4 with an onset at 145.4 ± 3.7 °C in the presence of CAT and at 154.7 ± 3.7 °C with an onset at 150.0 ± 4.2 °C in the presence of HEC, as quantitatively reported in Fig. 3c. Instead, integrating the peak to calculate the crystallization enthalpy (Δ*H*_c_), a different influence of the explored polymers on the crystallization process was observed, that increases from an average value of 639.9 ± 19.7 J g^−1^ for the MAPbI_3_ sample to 777.5 ± 1.8 J g^−1^ for CAT, up to 973.4 ± 74.5 J g^−1^ for HEC. In addition, a higher onset of evaporation temperature of DMSO was observed by TGA shown in Fig. 3c in the presence of the polymer with respect to the pristine perovskite, going from a Tonset of 100.4 ± 0.1 °C for pristine perovskite to about 119.2 ± 1.2 °C for CAT, until the highest around 129.4 ± 2.1 °C observed for HEC, as already reported in a previous study,^35^ confirming the need of higher energy to carry out the process.

The obtained results showed that the inclusion of different polysaccharides into the perovskite solution has a great impact on the crystallization process but in a different way.

Although the exact mechanism of crystallisation is still a subject of research, the LaMer theory, which qualitatively describes the nucleation and growth regimes by correlating the concentration of ‘precursor species’ as a function of reaction time, can be a useful model to combine the experimental results of the various characterisation studies to better understand what happens in our complex system. Indeed, the supersaturation, which according to this theory is the driving force of the crystallization process, is strictly related to the ‘removal’ of the DMSO after the precursor solution deposition. The delay in the DMSO evaporation influenced by the higher viscosity, and by the presence of the adduct (as evidenced by XRD analyses) induced after HEC and CAT inclusion, is evident and delays the crystallization peak in both formulations. The in solution interaction between the precursors and the functional group of the polymer, according to that observed by NMR analysis, is another factor of the system, which seems crucial to control the crystal growth. Indeed, a narrow and intense crystallisation peak was observed in the presence of HEC, where the combination of a higher viscosity and stronger interaction show a higher enthalpy which indicates greater stability of the film. This involvement in the perovskite crystallisation process has a first outcome in perovskite morphology. Compared to a reference perovskite morphology (Fig. 3d), the SEM analysis of the 30 MAPbI_3_–2.5 HEC film showed closely packed grains organized in the Liesegang rings (Fig. 3e). The formation of these particular structures usually occurs in gels where the nucleating particles induce a drop in the super-saturation levels of their surroundings, leading to the formation of spacing regions between nucleation centres.^46^ Instead, a broader peak was observed in the pristine MAPbI_3_ and in the presence of CAT, from which a lower enthalpy of reaction is calculated. The weakest interaction in solution between the MAI and CAT (observed for the explored concentration) results in a nonhomogeneous morphology, characterized by needle-like structures, as shown in Fig. 3f, which may be due to the imbalance between nucleation and growth rate. Additional Fourier Transform Infrared (FTIR) spectroscopy analyses were performed in Attenuated Total Reflectance (ATR) mode to analyse the presence of interactions in the film. No significant hydrogen bonding interaction between the –OH and methyl groups of the precursors was detected with this technique at the concentrations studied (Fig. S2†), while the shift of the C=O stretching vibration peak in the CAT from 1741 cm^−1^ (in powder) to 1754 cm^−1^ observed in the 30 MAPbI_3_ – 2.5 CAT (Fig. S3†) suggests the possible interaction of the carbonyl group with uncoordinated Pb^2+^ ions through the Lewis acid–base reaction.^71^ Nevertheless, it is worth underlining that the performance of CAT-based solar cells is dominated by the formation of perovskite films with discontinuous morphology, loaded of bundles and voids, so the possible passivation effect of the carbonyl on lead is marginal.

**Fig. 4:**
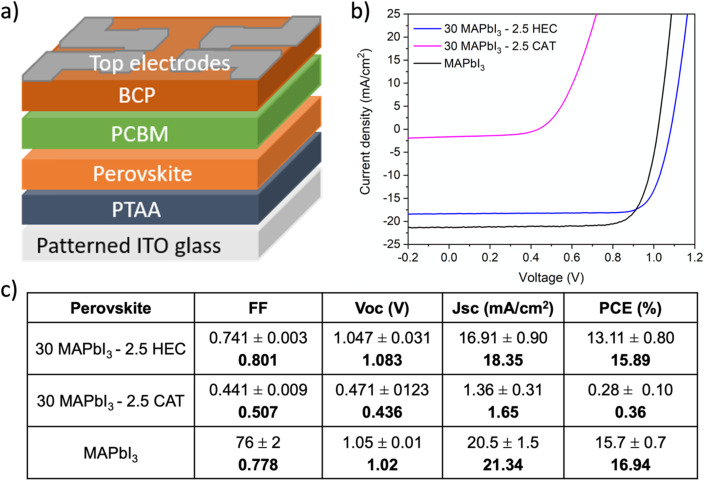
Photovoltaic parameters of the PPnc based PSCs. (a) p–i–n Device architecture and (b) characteristics *J*–*V* of the best device based on 30 MAPbI_3_ – 2.5 HEC, 30 MAPbI_3_ – 2.5 CAT and reference MAPbI_3_. (c) Average values (on 30 devices) and best performance (bold font).

These results highlight that in this complex system, the combination of a strong interaction with perovskite cations, together with the influence on the rheological behavior of the solution, appropriate selection of the polymer allowed developing stable inks that can be straightforwardly deposited in a single coating step at mild temperature, without the use of antisolvent bathing or dripping. Consequently, taking into account the diverse influence of the polymers, different device performances are achievable once integrated the perovskite based on HEC and perovskite based on CAT, as visible from Fig. 4.

After optimizing the fabrication conditions, 30 MAPbI_3_–2.5 HEC based solar cells displayed a maximum PCE of 15.89% with a high *V*_oc_ of 1.08 V and FF of 0.80, values comparable with those obtained using a reference MAPbI_3_ deposited with antisolvent dripping (Fig. 4b and c), demonstrating a compact and uniform film morphology with a net interface which minimizes shunting pathways and detrimental effects on photo-charge generation and collection processes.^22,35,72,73^ The good short circuit current density (*J*_sc_) of 18.35 mA cm^−2^ is adequate for the lower thickness of the 30 MAPbI_3_–2.5 HEC composite, around 220 nm, demonstrating again that the insulating nature does not interfere hugely with the charge transport in the active layer. Differently, the 30 MAPbI_3_–2.5 CAT based solar cells showed very low performances, around 0.36% resulting from the poor quality of the film. Therefore, proper engineering of the composite, in terms of selection of structure and functional groups of the polymer as well as the concentration and fabrication parameters, is required to have a beneficial effect of polymer deployment in the perovskite photoactive layer. A strong hydrogen bond between the polymer and the organic cation appears to be a necessary condition during the anti-solvent free crystallization process to properly drive the formation of perovskite, creating a homogeneous and high-performing film. Due to the poor morphology and performance the 30 MAPbI_3_–2.5 CAT has been excluded by further characterization.

In our previous work, we studied in detail the thermal stability induced by the presence of the HEC in the perovskite film and demonstrated a superior tolerance to thermal stress compared to a pure perovskite, mainly due to the interaction with the methylammonium, which physically prevents its sublimation as long as the polymer surrounds the perovskite grains.^35^ Thus, to further test the role of HEC as an effective intrinsic encapsulant of perovskite, the stability of the composite to light radiation by light/dark cycles was investigated. The films underwent an *in situ* aging process while performing real time EDXR measurements to monitor bulk and interface modifications. The samples were subjected to light/dark cycles alternating 8 hours of light (solar simulator 1.5 AMD, nitrogen controlled atmosphere) and 8 hours of dark, for an overall period of 64 hours. AFM and XRD analyses were also conducted to further evaluate the morphological and structural features of the samples.

**Fig. 5:**
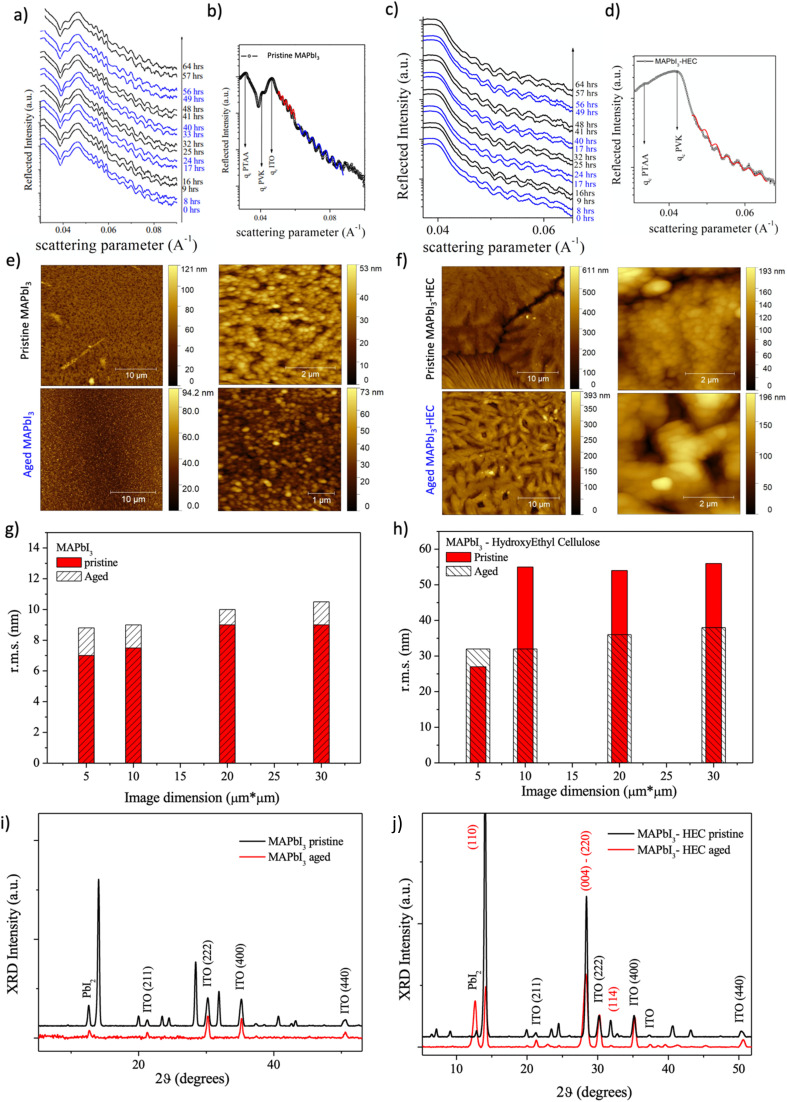
EDXR, AFM and XRD characterization. EDXR measurements during dark–light cycles for (a and b) reference MAPbI_3_ and (c and d) 30 MAPbI_3_ – 2.5 HEC; AFM analysis on (e) Reference MAPbI_3_ and (f) 30 MAPbI_3_ – 2.5 HEC, of pristine (top row) and aged (bottom row) samples, with relative (g and h) rms evaluation; XRD patterns of (i) reference MAPbI_3_ and (j) 30 MAPbI_3_ – 2.5 HEC before and after the aging process.

Fig. 5a reports time evolution of EDXR spectra (an extract) collected upon the reference MAPbI_3_ film during the light/dark aging process. Blue experimental data correspond to the first and last EDXR patterns collected during illumination, while the black one corresponds to dark exposure.

In Fig. 5b the pattern collected upon the pristine film is shown together with Parratt Fit of two different Kiessig fringes trends: perovskite morphological characteristics are obtained by the red fit, while the ITO fringes are fitted in blue.

As clearly visible, morphological parameters relative to the perovskite remains stable upon four light/dark cycles (overall 64 hours), with thickness and roughness being stable: *d* = 247.0 (5) nm; *s* = 0.55 (5) nm. The same observation can be made for the ITO substrate, with the evaluated thickness and roughness being *d* = 130.0 (5) nm and *s* = 0.40 (5) nm respectively. The same *in situ* aging experiment was performed on the 30 MAPbI_3_–2.5 HEC film, and the so obtained results are reported in Fig. 5c and d. The sample turns out perfectly stable from an EDXR point of view, with no thickness (*d* = 222.0 (5) nm) or interface/surface roughness (*s* = 1.0 (5) nm) variation being observed.

Morphological and structural characteristics of the samples, before and after light/dark cycling aging, were deduced by means of AFM and XRD to compare the texture and structure of the aged samples with their pristine state in the presence of HEC in comparison to the reference MAPbI_3_ perovskite only.

Before aging, pristine perovskite exhibits homogeneous texture with typical globular shaped formations as expected (Fig. 5e, top row). When HEC is added to the perovskite, big islands are formed with deep boundaries between the different regions (Fig. 5f, bottom row). Upon these islands the globular perovskite structure is still detected. However, several portions of the samples are also characterized by an elongated striped texture. A statistical evaluation of the root mean square (rms) roughness was performed and the results comparing pristine and 30 MAPbI_3_–2.5 HEC are presented in Fig. 5g and h, respectively.

The reference sample with pristine perovskite only evidences the lowest surface roughness mean value and the lowest rms dispersion (rms = 8.0 ± 0.4 nm; 6% dispersion). The value increases in the case of 30 MAPbI_3_ – 2.5 HEC (rms = 48 ± 14 nm; 29% dispersion).

After aging, the perovskite characteristic globular shape is retained in the reference perovskite, but a slight roughness increase is observed. Meanwhile, the surface texture of 30 MAPbI_3_ – 2.5 HEC is completely changed after the aging process. Indeed, in the case of the perovskite nanocomposite, the toughness is strongly reduced, and texture becomes very homogeneous independently on the image dimension. In the 30 MAPbI_3_–2.5 HEC film, perovskite globular aggregates are still visible, and the islands/stripes observable before the aging present elongated twisted fibers with the perovskite spherical aggregates superimposed.

In Fig. 5i the pristine (black line) *vs.* aged (red line) XRD patterns collected upon the reference MAPbI_3_ system are reported. After prolonged aging only ITO substrate reflections (International Center for Diffraction Data ICDD card nr: 00-06-0416) and PbI_2_ reflections (ICDD card nr: 00-001-0608) are retained, with the latter evidencing a 70% crystallinity loss. The crystalline signature of tetragonal MAPbI_3_ (space group *I*4/*mcm*, *a* = 8.800 Å, *c* = 12.685 Å) is lost suggesting that perovskite is strongly degraded into an amorphous structure. Indeed, no morphological thickness variation was observed during *in situ* EDXR measurements. The crystalline to amorphous transition process of the reference perovskite is also compatible with the slight surface roughness enhancement revealed by AFM analysis. PTAA contribution is still amorphous, thus not evidenced by XRD. Finally, XRD analysis conducted on 30 MAPbI_3_ – 2.5 HEC is reported in Fig. 5i: patterns of the pristine (black line) and aged (red line) film are compared. When the aged film is considered, ITO crystalline signals and PbI_2_ reflections are still visible (previously discussed), the latter signal showing evidence of crystallization (enhanced peak intensity 770%). Tetragonal MAPbI_3_ is still visible, however a strong crystallinity loss is observed for the perovskite structure, namely an 80% crystallinity loss along the (110) direction and a 50% crystallinity loss when the (220)–(004) combined reflection is considered, suggesting the [110] direction is mostly affected. Still no signal related to PTAA was observed. These experimental observations agree with EDXR results, supporting the evidence that 30 MAPbI_3_–2.5 HEC film thickness is retained during the aging process, and the light/dark cycling affects only perovskite crystallinity and not the overall film mechanical stability.

## Conclusion

The engineering of perovskite-based inks presents a valuable strategy to control both the crystallization process, and the stability of thin films fabricated using an antisolvent-free deposition method.

In this study, we investigated the role of the –OH functional groups present in polysaccharides in the crystallization dynamics and stability of perovskite thin films. By comparing HEC and CAT as polymeric additives in 30 MAPbI_3_, we demonstrated that the nature and strength of polymer–perovskite interactions significantly influence film formation, morphology, and long-term stability.

NMR analysis revealed that HEC establishes strong hydrogen bonds with perovskite precursors, particularly with methylammonium ions, which in turn affects the crystallization process by modulating the nucleation and growth of the perovskite phase. This interaction was further supported by DSC and TGA measurements, which showed a delayed crystallization peak and a higher enthalpy of crystallization in the presence of HEC, indicating a more controlled and thermodynamically stable film formation process. Conversely, CAT, with partially substituted hydroxyl groups, exhibited weaker interactions, leading to reduced control over perovskite crystallization and poorer film quality.

Morphological and structural characterization further confirmed the beneficial effect of HEC on film formation. SEM and AFM analyses revealed that HEC-based perovskite films exhibited a highly uniform grain structure with the formation of Liesegang rings, indicative of a self-assembled growth mechanism driven by the polymer's interaction with perovskite precursors. In contrast, CAT-based films showed needle-like structures and poor crystallinity.

The improved morphological and structural properties of HEC-based perovskite films directly translated into enhanced photovoltaic performance. The solar cells incorporating MAPbI_3_-HEC achieved a power conversion efficiency of 15.89%, comparable to standard antisolvent-processed perovskites, demonstrating that HEC does not compromise charge transport properties while promoting a more stable and defect-resistant film. While MAPbI_3_-CAT-based solar cells exhibited extremely low efficiencies (∼0.36%) due to poor film morphology and high defect density.

To further evaluate the role of HEC as an intrinsic stabilizer, we conducted an aging study under light/dark cycles, monitoring bulk and interface modifications through energy dispersive EDXR, AFM, and XRD. EDXR analysis confirmed that the thickness and roughness of MAPbI_3_-HEC films remained stable after prolonged exposure to light, whereas pristine MAPbI_3_ films underwent significant degradation. AFM measurements showed that, although HEC-based films initially had a higher roughness than pristine perovskite, their morphology evolved over time into a more compact and homogeneous texture, suggesting a stabilization effect. XRD data further supported this conclusion, revealing that while both MAPbI_3_ and MAPbI_3_-HEC films experienced crystallinity loss upon aging, the structural degradation was far more pronounced in the pristine perovskite, which converted almost entirely into an amorphous phase and PbI_2_.

These findings highlight the crucial role of polymeric additives in modulating perovskite crystallization and stability. By tailoring the functional groups of polymer-based additives, it is possible to develop robust and processable perovskite inks that enhance film durability without the need for complex processing steps. Future research should explore a broader range of polysaccharides with varying functional moieties to further optimize perovskite crystallization and stability, paving the way for scalable and long-term stable perovskite solar cells.

## Ethical approval

Ethical approval is not applicable to this article as no experiments involve human tissue.

## Data and availability

The data that support the findings of this study are available from the corresponding authors upon reasonable request.

## Author contributions

Conceptualization: A. R. and A. G.; methodology: A. R. and A. G.; validation: A. R., A. G., and R. P., P. B.; formal analysis: F. B., S. C., S. S., A. Ge., M. G.; investigation: F. B., S. C., S. S., M. G.; writing—original draft preparation: F. B., A. G.; writing – review &editing: F. B., A. G., R. P., P. B., B. P., A. L., S. Co., A. R.; supervision: A. G., C. E. C., A. R.; project administration: A. R. All authors have read and agreed to the published version of the manuscript.

## Conflicts of interest

The authors declare that they have no known competing financial interests or personal relationships that could have appeared to influence the work reported in this paper.

## Supplementary Material

NA-OLF-D4NA01036A-s001
