# Supplementary material for: Inclusion of polysaccharides in perovskite thin films: from in-solution interaction to film formation and stability
Source: Nanoscale Adv. 2025 Mar 11;7(8):2145–57. doi: 10.1039/d4na01036a (PMC11913129; doi:10.1039/d4na01036a)
Supplement: NA-007-D4NA01036A-s001 [file NA-007-D4NA01036A-s001.pdf]

Supplementary Information (SI) for Nanoscale Advances.
This journal is © The Royal Society of Chemistry 2025

**Supporting information**

# Inclusion of polysaccharides in perovskite thin film: from in-solution interaction to film formation and stability

Francesco Bisconti,[1] Antonella Giuri,[1*] Nadir Vanni,[1,2] Sonia Carallo,[1] Silvia Spera,[3] Rosamaria Marrazzo,[3] Riccardo Po',[3] Paolo Biagini,[3] Barbara Paci,[4] Amanda Generosi,[4] Marco Guaragno,[4] Carola Esposito Corcione,[5] Andrea Listorti,[6] Silvia Colella,[7] Aurora Rizzo[1*]

1 CNR NANOTEC – Istituto di Nanotecnologia, c/o Campus Ecotekne, Via Monteroni, 73100 Lecce, Italy;

2 Dipartimento di Matematica e Fisica "E. De Giorgi", Università del Salento, Campus Ecotekne, via Arnesano, 73100 Lecce, Italy;

3 Research Center for Renewable Energy & Environmental, Istituto Donegani, Eni S.p.A., via Fauser 4, I-28100, Novara, Italy;

4 CNR-ISM- SpecX Lab Via del Fosso del Cavaliere 100, Roma

5 Dipartimento di Ingegneria dell'Innovazione, Università del Salento, via per Monteroni, km 1, 73100, Lecce, Italy;

6 Dipartimento di Chimica, Università di Bari, Via Orabona 4, 70126 Bari, Italy;

7 CNR NANOTEC – c/o Dipartimento di Chimica, Università di Bari, Via Orabona 4, 70126 Bari, Italy;

Corresponding author: antonella.giuri@nanotec.cnr.it & aurora.rizzo@nanotec.cnr.it

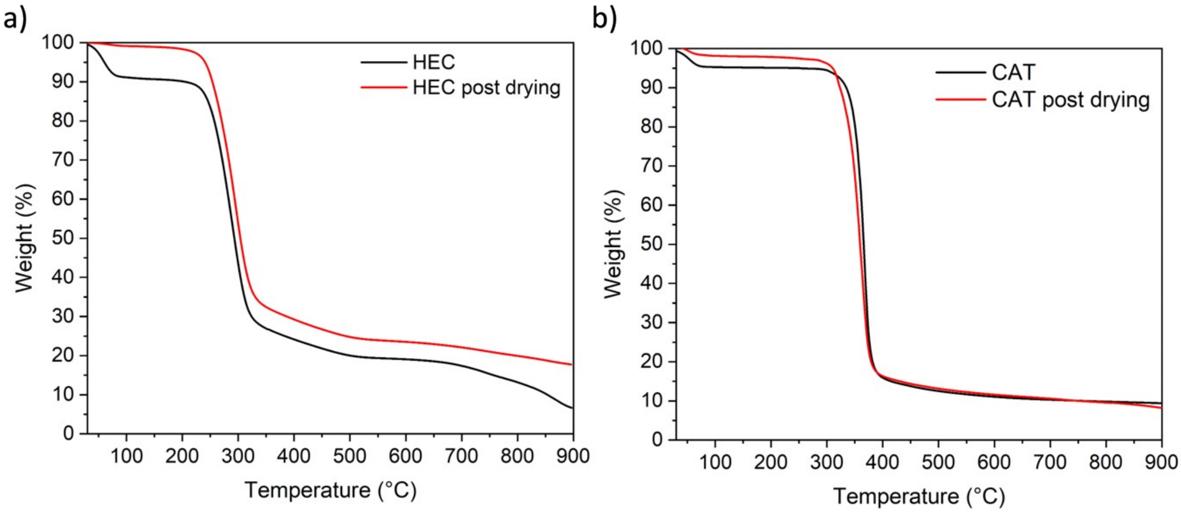


**Fig. S1. TGA analysis of polymer before and after drying process.**

**Tab. S1. TGA analysis of polymer before and after drying process.**

| Polymer | I step | | II step | |
|---|---|---|---|---|
| | Weight loss wt % | $T_{endset}$ °C | Weight loss wt % | $T_{onset}$ °C |
| **HEC** | 9.2 | 86.3 | 72.2 | 255.9 |
| ***HEC post drying*** | *1.0* | *81.8* | *75.5* | *256.3* |
| **CAT** | 4.7 | 68.8 | 85.3 | 350.7 |
| ***CAT post drying*** | *2.2* | *69.9* | *87.0* | *336.8* |

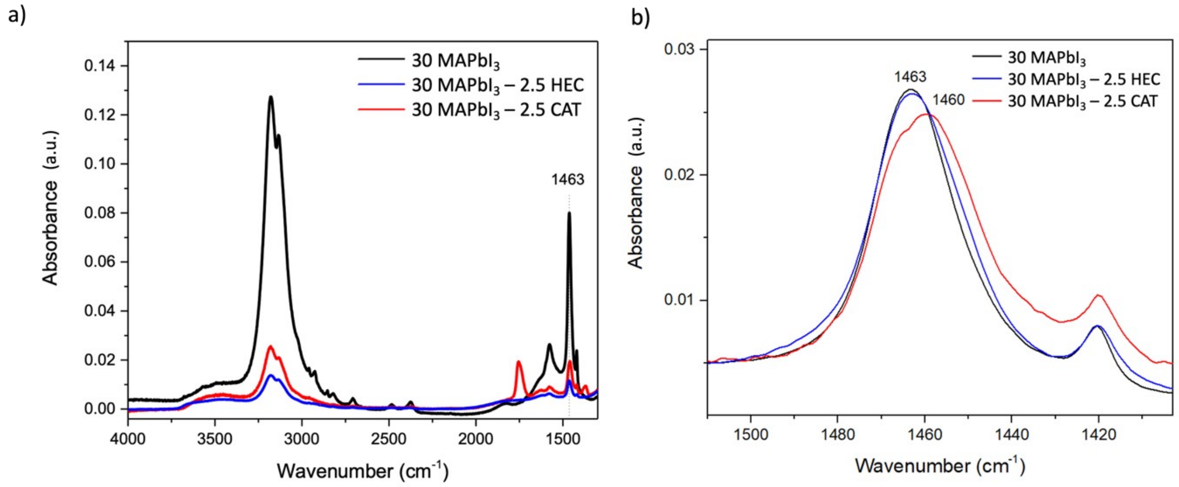


**Fig. S2. FTIR-ATR spectra of a) pristine $MAPbI_3$, 30 $MAPbI_3$ – 2.5 HEC and 30 $MAPbI_3$ – 2.5 CAT, with b) magnification of the N-H bending (spectra normalized to -CH3 bending 1422 $cm^{-1}$.**

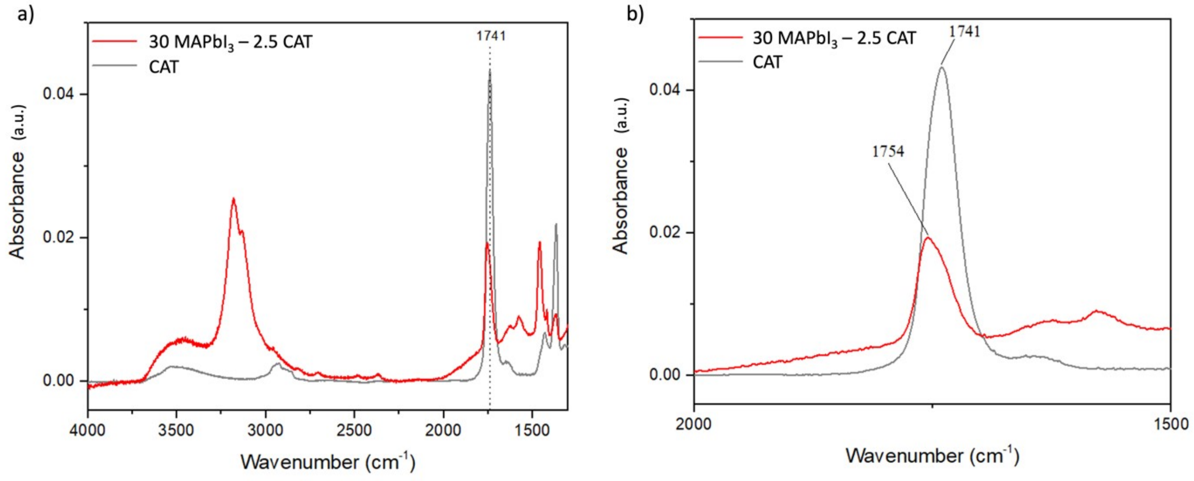


**Fig. S3. FTIR-ATR spectra of a) 30 $MAPbI_3$ – 2.5 CAT and CAT samples, along b) a magnification of the region in which fall to the C=O stretching.**
